# Stool submission by general practitioners in SW England - when, why and how? A qualitative study

**DOI:** 10.1186/1471-2296-13-77

**Published:** 2012-08-08

**Authors:** Cliodna AM McNulty, Gemma Lasseter, Katie Newby, Puja Joshi, Harry Yoxall, Kalyanaraman Kumaran, Sarah J O’Brien, Mark Evans

**Affiliations:** 1 Health Protection Agency, Primary Care Unit, Microbiology Department, Gloucestershire Royal Hospital, Great Western Road, Gloucester, GL1 3NN, UK; 2 Applied Research Centre in Health and Lifestyle Interventions (ARC-HLI), Faculty of Health & Life Sciences, Coventry University, Priory Street, Coventry, West Midlands, CV1 5FB, UK; 3 Blackbrook Surgery, Taunton, TA1 2LB, UK; 4 Communicable Disease Control, South West (South) Health Protection Unit, Exeter, EX1 3QS, UK; 5 Infection Epidemiology and Zoonoses, Institute of Infection and Global Health, University of Liverpool, Neston, CH64 7TE, UK; 6 South West (North) Health Protection Unit. Health Protection Agency, Bristol, BS1 6EH, UK

**Keywords:** Stool specimens, Microbiology, Laboratory submission, Diarrhoea, Primary care, Qualitative, National guidance

## Abstract

**Background:**

We know little about when and why general practitioners (GPs) submit stool specimens in patients with diarrhoea. The recent UK-wide intestinal infectious disease (IID2) study found ten GP consultations for every case reported to national surveillance. We aimed to explore what factors influence GP’s decisions to send stool specimens for laboratory investigation, and what guidance, if any, informs them.

**Methods:**

We used qualitative methods that enabled us to explore opinions and ask open questions through 20 telephone interviews with GPs with a range of stool submission rates in England, and a discussion group with 24 GPs. Interviews were transcribed and subjected to content analysis.

**Results:**

Interviews: GPs only sent stool specimens to microbiology if diarrhoea persisted for over one week, after recent travel, or the patient was very unwell. Very few had a systematic approach to determine the clinical or public health need for a stool specimen. Only two GPs specifically asked patients about blood in their stool; only half asked about recent antibiotics, or potential food poisoning, and few asked about patients’ occupations. Few GPs gave patients advice on how to collect specimens.

Results from interviews and discussion group in relation to guidance: All reported that the HPA stool guidance and patient collection instructions would be useful in their clinical work, but only one GP (an interviewee) had previously accessed them. The majority of GPs would value links to guidance on electronic requests. Most GPs were surprised that a negative stool report did not exclude all the common causes of IID.

**Conclusions:**

GPs value stool culture and laboratories should continue to provide it. Patient instructions on how to collect stool specimens should be within stool collection kits. Through readily accessible guidance and education, GPs need to be encouraged to develop a more systematic approach to eliciting and recording details in the patient’s history that indicate greater risk of severe infection or public health consequences. Mild or short duration IID (under one week) due to any cause is less likely to be picked up in national surveillance as GPs do not routinely submit specimens in these cases.

## Background

One in five of the population in England suffer each year from Infectious Intestinal Disease (IID) with over 1,800 deaths and 53,000 hospital admissions in England and Wales reported during 2009/10 [1,2]. However, the majority of episodes are often mild and self-limiting, with the most common symptoms being diarrhoea and vomiting.

The majority of information submitted to surveillance systems in the UK is obtained from stool specimens submitted from Primary Care to microbiology laboratories. The Public Health (Control of Disease) Act 1984 requires cases of suspected food poisoning to be reported by any medical practitioner to their local Health Protection Unit [3] and since the early 1980s food poisoning notifications have steadily risen [4]. The investigation and reporting of foodborne outbreaks is mandatory within the European Union (EU) (Directive 2003/99/EC). The Health Protection Agency (HPA) has created an electronic Foodborne and Non-foodborne Gastrointestinal Outbreak Surveillance System (eFOSS), which generates a national dataset [5] on the severity of disease, the importance of different agents, food types and factors contributing to the occurrence of foodborne outbreaks. The incidence of IID in the UK is routinely underestimated by these national statistics, because members of the public often fail to present to their general practitioner (GP) for investigation [6]. The recent IID2 study estimated that for every 147 community cases of IID, only ten visited their general practice and only one case was reported to national surveillance [6]. Although the IID2 study determined the burden of IID and calibrated national surveillance systems in the United Kingdom, it did not investigate what criteria GPs use to decide when to send a stool sample and whether any national guidance informs their decision making.

In 2007 the Health Protection Agency (HPA) and British Infection Association (BIA), in consultation with primary care stakeholders, produced guidance for primary care practitioners on when and how to submit stool specimens for microbiological investigation, how to interpret the stool report, and when repeat specimens should be sent [7]; this forms the basis of the Clinical Knowledge Summary (CKS) guidance [8]. This guidance is available in electronic format on the HPA and CKS websites.[7,8] There are also web links to this guidance from the HPA antibiotic guidance which is used by over 70% of PCTs in England to develop their local guidance; however the web links are not always present in local adaptations of guidance. We do not know if GPs use, know of, or value the HPA or CKS guidance or other guidance, or if they follow their recommendations.

The aims of this audit and service evaluation were to determine what criteria GPs use to decide when to send stool samples, what (including national guidance) informs these decisions, and their opinions of the national guidance available. As there has been very little work in this area we used qualitative methods in order to obtain a range of opinions using open questions. The results will give an indication of adherence to the guidance by GPs, inform future review and provision of and accessibility to national guidance and inform reasons for the bias in surveillance figures for IID. In addition, this work will enable the development of a questionnaire survey of a larger number of GPs.

## Methods

We collected data through telephone interviews with general practitioners from surgeries with a range of stool submission rates, and through a discussion group held at an educational event to discuss stool submission.

### Participants

For the telephone interviews, 182 general practice surgeries served by three microbiology laboratories in three areas in SW England (Gloucester, Taunton and Salisbury) were identified via the laboratory computer systems. Surgery populations ranged from 630 to 17,102 (mean surgery population 7,210) patients. Surgeries were ranked in order of their stool submission rates per 1000 patients in 2009 and stratified into lower (9/1000 patients), average (15/1000 patients) or higher mean stool submissions (24/1000 patients). Surgeries from each group were then listed randomly and contacted via telephone by CMcN in order from the top of this list. The duty doctor on call at each surgery on the day of the initial telephone call was invited to participate in a telephone interview at a later date regarding their stool specimen submission practices. All willing participants were sent a study information sheet and consent form by post or email. Upon consent GPs were sent a copy of the Primary Care Infectious Diarrhoea Guideline produced by the HPA, but were specifically asked not to view this document prior to interview. All surgeries were offered £50 as compensation for their GP’s time.

To enrich the data on GPs’ opinions on the HPA guidance, a GP discussion group was organised, in collaboration with the Severn Deanery GP School, and presented as an evening educational talk. The session focused on “Tips and Common Pitfalls in GI investigations” and was presented in April 2011 at the Nuffield Health Cheltenham Hospital. The evening was attended by a convenience sample of 22 GPs, from 19 surgeries throughout Gloucestershire. None of the GPs at the study evening were involved in the interviews.

### Interview schedule and procedure

A semi structured interview schedule (Additional file 1: Appendix A) was developed for the telephone interviews by researchers and was informed by the study objectives, literature and HPA guidelines for stool specimen submission. The schedule investigated attitudes towards stool specimen microbiology, decision making around submission, processes followed in submitting specimens, attitudes towards the value of reports, and the clinical management of patients presenting with diarrhoea (Table 1). Two pilot interviews were conducted with GPs in Somerset, however no amendments were required.

**Table 1:** Reason given by GPs interviewed (from lower, average and higher stool submitting surgeries) for sending a stool specimen for microbiology laboratory investigation

	**Stool submission rate (number of GPs)**		
**Criteria**	**Lower (n = 5)**	**Average (n = 6)**	**Higher (n = 9)**
Duration of illness	5	5	7
Travel abroad	3	2	3
Generally unwell	1	2	3
Blood	1	2	1
Exclude infection	1	2	4
Chronic illness	1	-	-
Anxious	1	-	1
Recent Antibiotics	1	-	1
Family members ill	1	-	1
Frail patient	1	-	-
Systemic symptoms	1	-	-
Quality of stool	-	-	1

Telephone interviews were conducted by two researchers (KN and CB) via telephone, between June and August 2010. The interviewers were trained in qualitative interviewing techniques and discussed how to prevent bias before and after the pilot interviews, they had no affiliation with the HPA and were neutral to the aims of the study. Interviews lasted 25 to 40 minutes, were recorded digitally and transcribed verbatim. Data was anonymised using a coding system i.e. GP1, GP2. All participants were sent a transcript of their interview in the post and were asked to correct/comment on the transcripts. Only one GP made a minor spelling change. Following the first two interviews, the interview schedule was reviewed by the research team to determine the quality of data being generated. However no changes were required.

### Discussion group schedule and procedures

An educational talk was developed for the discussion group and structured as a PowerPoint presentation, which reviewed the HPA primary care infectious diarrhoea guidance (Additional file 2: Appendix B). Each section of the guidance was presented to the group and reviewed separately. Participants were prompted to discuss the content, layout and whether they agreed with the information presented. The questions in the interview schedule that asked about the guidance were also asked of discussion group participants.

The discussion group was conducted by a researcher who was a member of the HPA and had extensive knowledge of the HPA guidance and personal experience of processing stool specimens from GPs. Her knowledge allowed her to facilitate an informed discussion about the contents and layout of the guidance. The discussion group lasted 30 minutes and was digitally recorded and transcribed verbatim.

### Data analysis

Data were anonymised using a coding system i.e. GP1, GP2 and DG1, DG2 etc. Data from the telephone interviews and discussion group were subjected to content analysis [9]. Analysis of interview data was performed first and carried out by university researchers (PJ and KN). Data analysis employing deductive content analysis takes a ‘top down’ approach with preliminary codes developed before data analysis begins. In this study, codes were informed by the audit objectives, and the content of the HPA guidance. Twenty-seven codes were identified (Additional file 3: Appendix C), with operational definitions for each code developed by researchers and agreed upon by a steering group. Use of NVivo software (QSR International PTY Ltd Melbourne) facilitated organisation of the data. Interview analysis was performed by two researchers. The first researcher read and re-read each interview transcript to achieve an overall understanding of its content. Transcripts were then analysed individually by annotating words and sentences (a unit of analysis) and assigning relevant preset codes to each. This was an iterative process with transcripts reviewed a third time and adjustments made if necessary. A second researcher coded 10% of the data for reliability and quality assurance. Discrepancies were resolved through discussion between the researchers and with the steering group. The frequency of each unit of analysis was recorded allowing researchers to identify the most frequent responses, providing an indication of the most salient points.

The discussion group was subjected to a similar content analysis based only on data generated from questions about the guidance which were shared by both the interview and discussion group schedules (see Additional file 1: Appendix A). The data was analysed by GL and reviewed with CMcN.

### Ethics

This study was considered to be an audit and NHS ethical approval was not required. The study was given ethical approval by Coventry University. Clinical governance approval was obtained from Gloucestershire, Somerset and Wiltshire PCTs.

### RATS

This study adheres to the RATS guidelines on qualitative research.

## Results

### Interview participants

Of the 25 GPs initially contacted, five said they had insufficient time to participate, 20 (80%) agreed to participate in a telephone interview. GPs were recruited from five low stool submitting surgeries, six average and nine high (mean submission 9/1000, 15/1000 and 24/1000 patients respectively). Sixty-five percent of participants were male, the mean GP age was 49 and the median number of years since qualifying was 24 (range: 10–36 years). The mean number of partners in each participating surgery was four and the mean number of sessions worked by each participating GP per week was seven. Nine surgeries were in a suburban area, seven were rural and four were inner-city. Fifty five percent of GPs had a personal list and 11 of the surgeries were training surgeries. Two of the participating GPs provided Gastroenterology sessions at their local hospital; all other participants had no specific clinical interest in GIT diseases.

### Discussion group participants

Twenty two GPs from 19 surgeries throughout Gloucestershire attended the discussion group. Where they worked at a single surgery, GPs were from 11 low, two medium and three high stool submitting surgeries.

### Interview results

There were no themes that were characteristic of low, average or high stool submitting surgeries and therefore the data for the three categories has been combined. The most important data are presented, with verbatim quotes to support selected themes, in Tables 2, 3, 4, 5, 6, 7, 8, 9, 10.

**Table 2:** Value of stool specimen microbiology to GPs in patients with diarrhoea

**Attitude/Opinion**	**Quotes**
Stool microbiology useful	GP16 *“I think it’s a positive thing actually.”*
	GP13 *“It’s useful in two situations: prolonged diarrhoea or diarrhoea followed from abroad”*
	GP2 *“Yeah I do, actually think it’s important because about 1 in 10, 1 in 20 come back with something that we should know about. And the other thing is the old thing about the patients being reassured that it isn’t something that we need to treat”*
	GP9 *“Helpful in certain settings. So very much on return from travel, very much if there’s systemic symptoms and if it’s prolonged over a few days, then I would find it helpful and reassuring”*
Usually treat before result received	GP7 *“Well when we send them they’re usually useful but more retrospectively useful. You usually have to make a decision whether you’re going to treat somebody or not before getting the sample back. I think they’re a good thing.”*
	GP1 *“Depends how they are. A campylobacter would be the main one. Someone presenting with bloody diarrhoea and pain, who we think probably isn’t an inflammatory bowel disease, you then do a stool sample and. I might wait for the samples or I might just get on and treat them”*
Stool microbiology not useful	GP14 *“Not necessarily very helpful. I might, so generally speaking I wouldn’t send a specimen. Because most tend to be self-limiting. This is I’m thinking about acute infectious diarrhoea, most tend to be self limiting and are not likely to be treated actively.”*
	GP3 *“For most cases, it’s of limited value because most diarrhoea illnesses are self-limiting.”*

**Table 3:** Most common reasons for sending a stool sample to microbiology in diarrhoea

**Participant**	**Quotes**
GP16	*“I would send microbiology samples if people had persistent diarrhoea, which would mean a week or more depending on the person.... If someone has presence of blood or systemic symptoms that’ll be the next best common.”*
GP5	*“Duration of diarrhoea, the general health of the patient, so if they are not quite right and that’s gone on for longer than a week. I tend to use one week as my guideline.”*
GP18	*“Yeah, I mean it’s really I suppose if I have a high index of suspicion that there’s going to be something there. So travel abroad would be an indication to do it quite soon probably. If it’s been going on for longer than a week I would probably do it. Again I guess if there was a sort of a concern that there might be a definite cause. So somebody might say well we’ve been out, we went out for a meal or we went for a barbecue and three of the family have got diarrhoea then I probably would send stool specimens in that situation.”*
GP20	*“If the diarrhoea has been going on for more than two weeks or if the patient is very ill.”*
GP10	*“The most one that I’ve done is for prolonged diarrhoea really.”*
GP13	*“There are only two real reasons, one is, the two commonest by far is prolonged diarrhoea or the diarrhoea following travel abroad.”* Interviewer**:** And for prolonged diarrhoea what would your definition be?
GP13	*“Over a week.”*
GP4	*“If there had been recent travel abroad, you know, particularly somewhere that had a higher infection risk. The duration of time it’s been going on for, and I suppose whether I knew that there was something going around that I’d seen a lot of people with, that would make me send a stool specimen perhaps more sooner than I otherwise might.”*
GP14	*“Often if symptoms are going on, you know, a week, ten days. If somebody’s been abroad and is unwell, systemically, they’re the main reasons really I would think.”*
GP17	*“Most commonly it would be an adult, although sometimes children if it’s very prolonged, but most commonly it’s adults with a longer than usual episode of diarrhoea or if they’re markedly unwell with it.”*
GP15	*“If the diarrhoea was very short term, unless there was an obvious thing that I was worried about I would, forgive me saying, just let it run. And yeah, I suppose if people are clinically well in themselves thinking about it and they say they’ve had some diarrhoea and you go through a careful history and there’s no obvious cause, I would probably let that be for four or five days before I sent a sample.*”
GP19	*“I would think probably someone whose diarrhoea isn’t settling. Is having it prolonged, for a prolonged period.”*

**Table 4:** Public health issues GPs’ discussed with patients

**Questions asked by GP**	**Quotes**
Do GPs ask about food related illness and occupation when patients have diarrhoea?	GP3 *“I’ll normally ask if they can think of any reason why this has occurred and if any other family members or people that they’ve been associated with have got it. And again if you’re seeing a clear association that you think may have a public health issue then I would be sending a stool sample.”*
	GP7 *“I sort of need to ask what job they do, are they involved in food handling at all or working in the food industry or in restaurants, and if they are you would probably need to advise them not to work until their symptoms have settled or until you’ve got a diagnosis”*
	GP8 *“I tend to ask them what their job is, and I always tell people if they’ve got diarrhoea they mustn’t be at work especially if they work with children or handle food within 48 hours of any symptoms.”*
Do GPs ask about other related cases when patients have diarrhoea?	GP4 *“Well I always ask them if anyone else in the household has got it, or I suppose quite often they volunteer the fact if they think they ate something dodgy, or I will say to them have you eaten anything unusual, I will usually say that”*
	GP19 *“Yes, I would, yeah. I’d ask them about that, if they’d eaten somewhere different or if anyone else in the household or in the group had similar symptoms”*
Do GPs ask about recent antibiotic use when patients have diarrhoea?	GP10 *“Not always I don’t think, no. I mean usually if it’s your patient you would know, you know, but I probably don’t to be honest. Probably should but don’t”*
	GP11 *“I do, yes. Particularly, we get quite a lot of temporary residents here in where we are in x; we get a lot of visitors here and I will always ask them if they’ve had anything recently. Obviously we have our medical records in front of us who can tell us they’ve had something pretty powerful recently”*
Do GPs ask about farm visits when patients have diarrhoea?	Interviewer: And what about things like young people having visited a farm recently, that kind of thing, would you ask about that?
	GP9 *“Probably not enough. Certainly we’ve got our fairly rural practices so I’d tend to ask about working on farms etc, but probably not ask enough about day visits. I mean usually some sort of question any ideas you know any thoughts of where it might have come from. So I’m quite interested in the patient’s individual beliefs about where things have come from. And that often an open question can pick up certain things.”*
	GP20 *“No, although I probably should. I might well do after the news a few months ago, the E. coli outbreak.”*
	GP18 *“No, I don’t specifically. But I take the point, probably worth doing. We do have one or two sort of open farms around here.”*

**Table 5:** When GPs send samples requesting Ova, cysts and parasites in patients with diarrhoea

**Participant**	**Quotes**
GP10	*"I suppose that if they’ve been abroad really, you know, and that’s really third world travel”*
GP7	*“Well I guess if someone’s come back from India or somewhere where you’d suspect they’ve picked up a parasitic infection. I guess if they work in sewers and things like that. I don’t know whether that would be a situation where you’d ask for that sort of test.*
GP13	*“Don’t think I ever would, I’d leave that up to the lab really"*
GP11	*“don’t actually but it comes back, I mean I’ve never asked for a specific thing. I think I may have asked for clostridium difficile once. But I’ve just sent a normal MC&S really. I don’t have much sort of sophistication in terms of exactly what I’m asking for I’m afraid.”*

**Table 6:** Process of obtaining stool specimens from patients with diarrhoea

**Participant**	**Quotes**
GP3	*“I mean obviously it can sort of, it’s not particularly pleasant, but most individuals don’t seem to mind if they think it’s appropriate. You know, if they’re unwell, then they’ll do it.”*
GP10	*“I fill in a black form, a microbiology form, and I give them a stool pot with a little spoon thing in it. And I say can you do a sample by fair means or foul and put it in this pot and then put it in the bag, seal it up and hand it in to surgery the same day.*
	Interviewer**:** Do you tell them how much of a sample to collect?
	***“****No, I don’t actually you know at all.”*
	Interviewer: Do you give them any advice on how to collect the specimen?
GP11	*“Physically? I give them a pot and I explain what the spoon is for, give them a little bit of a clue as to what they might find helps, you know, ice cream box cleaned out in the toilet, and wish them good luck really.”*
GP12	Interviewer: Do you tell them roughly how much of a sample to provide?
	“*No because I wouldn’t know.”*
GP20	*"I tell them they need to do three samples on separate days and that it needs to be taken to the lab preferably straight after the sample’s done.”*

**Table 7:** Suggested improvements to the stool submission process

**Participant**	**Quotes**
GP1	*“Well, there are, when we use the electronic version, there are cues that come up, so perhaps slightly better cues might be helpful.”*
GP10	*“Giving us a more detailed form. So tick boxes or, you know, saying how long have they had the diarrhoea, just questions that prompt, yes is there any blood you know?, have they got systemic illness?, are there any other .... concurrent illness going on?*

**Table 8:** Interpretation and value of the microbiology stool report by GPs

**Theme**	**Quotes**
Interpretation by GPs of a negative stool microbiology report	GP14 *For me a negative report would be something that had, you know, no culture positive result or no other abnormalities in the report, like ova’s or cysts or whatever.”*
	GP7 ***“****Well I’m assuming a negative report means to me a result where they haven’t found any of what they were looking for, any significant, if you're asking for rotavirus, they haven’t found that or they haven’t found any bacterial infections or they haven’t found any ova, cysts or parasites. So they haven’t found a specific infection there.”*
	GP8 *“A negative report means they haven’t grown anything, it doesn’t always mean there’s nothing there. Parasites, because you’ve got to wait for them too, … so it’s not 100%.”*
	GP9 *“That it’s not shown a specific sort of pathogenic bacteria,.... it’s not saying there isn’t a viral gastroenteritis; it’s … about the more significant ones that I need to act on. It’s not saying they’re not infected diarrhoea.”*
Value and Influence of the microbiology report on management of diarrhoea	GP6 *“Well, quite strongly. If say they’ve got something like campylobacter and you ring up and they say they’ve still got bad diarrhoea, then it was a good indication to give them some treatment”*
	GP1 *“It’s very useful. You know, if you’ve got, you know, to make the diagnosis, differentiate between say an infection or inflammatory bowel disease.”*
	GP19 *“Yeah, I mean they give negatives as well as positives. So it’s useful to be able to tell people they don’t have X, Y or Z, as well as if they have got a positive result. Yeah I mean I think they give all the information I need. Plus we have a very helpful microbiology department locally. So I guess if there was something or I was unsure about I would phone one of the consultants up at the hospital and just take some advice.”*
	GP11 *“It does and if anything come out of it there’s usually a piece of helpful advice as well about the significance of what it is or insignificance sometimes or whether to discuss with somebody else.”*
	GP3 “*Well, I don’t have any negative views about them. You know, they give all the information we need.”*
	Negative opinions about reports
	GP10 *“Well it’s* (the report) *very basic. I mean I’ve got one in front of me, it’s got final report, appearance, Bristol Type 1, and I can’t remember the types. I know Bristol Type 1 goes to probably one to six or something and its different stools. And then it’s got microscopy and that’s blank … and then it’s got culture and … it just says salmonella, shigella not isolated, campylobacter, E. coli 0157 not isolated – that’s it.*
	GP14 *“I think our positive pick up rate’s very low, and if we do generally pick something up then… their illness is often resolved. So they’re not necessarily that helpful.”*
Antibiotic treatment reported by GPs in cases of diarrhoea	GP12 *“Well I suppose if I had a fairly, if clinically I really felt that someone possibly had salmonella or campylobacter, clinically, then I might treat and get them to do samples and then review it. But that’s so unusual. My general, my default position is not to do anything until I know what I’m dealing with.”*
	GP13 *“In two situations, one would be a positive result from the lab and the second would be bloody diarrhoea having travelled abroad, which case I’d give them erythromycin. .... I’d expect them to provide a stool specimen and then start the antibiotics whilst waiting the result, yeah.”*
	GP18 *“Well I hope I’ve got that information. I don’t particularly say to the patient, I suppose I ought to in case they’ve got antibiotics from somewhere else, but I generally, I would make an, you know, I would look at their medication and look at what they’d had recently certainly. So I would take it into account. But I don’t suppose I ask specifically have you been anywhere else and got antibiotics.”*
	GP19 *“If it’s our patients I would probably be aware of that anyway. So I’m not sure I’d necessarily ask; I would look on their notes and see if they were on antibiotics or has recently had a course*.”
	GP7 *“Well I think, as I say, you often have to make a decision about whether to treat with antibiotics at the time you’ve decided that you’ll obtain a sample. Occasionally if it’s very mild, you’ll hold off treating until you’ve got the result back, but sometimes those symptoms are quite* [severe] *so that you’ve already made a decision to treat with antibiotics, and so you’re using the sample then just to confirm the course of action that you’ve taken three days before, but it’s still useful just to confirm you have treated them correctly, and then you can take the resolution symptoms to confirm that that infection has cleared.”*

**Table 9:** Sources of advice for GPs on when to submit stool samples

**Participant**	**Quotes**
GP7	*“Phone microbiology or I’d look on the pathology website.”*
GP14	*“Well I’d probably need to ring up one of the microbiology consultants to ask whether they thought that was a valuable investigation to do, and they’re usually very easy to get a hold of.”*
GP17	*“It’s unusual for me to be in that position, I have to say, so if there was a particular concern it might be more in terms of do I need to look for something fairly easily taken, I’d probably ring up the laboratory.”*
GP3	*“I would rarely look for guidance as about sending stool samples because it’s, you know, there’s not easy access to clear information about that, and it’s really on clinical judgment, and if I had doubt I would send one. So I don’t think I’ve ever looked up about appropriateness of sending a sample in a given situation.”*

**Table 10:** Usefulness of stool specimen guidance to GPs in patients with diarrhoea

**Participant**	**Quotes**
GP1	*“It might be useful if they added on to the bottom of the reports ‘Please see such and such for up to date guidance on stool specimens or whatever.”*
GP11	*“Some written guidance is always helpful. I’m quite sort of electronic, so I think electronic, the intranet really, or the internet.”*
GP7	*“I think I’d want that as guidance that we could maybe scan onto the computer onto the protocol part of the computer, and we could look it up..... We have various protocols of that sort … on the computer anyway. … The fact that they’re on the computer means I can access them quite easily. I don’t have to remember where I’ve put the bit of paper.”*

### Value of stool specimen microbiology

The majority of interview participants (17), reported stool specimen microbiology was a useful and positive process but submission must be based on the individual patient’s symptoms, history and needs. However, over half of the GPs (11) reported that they usually had to make a decision whether they were going to treat somebody or not before getting the report back; this was mainly when they suspected diarrhoea due to campylobacter. Three GPs felt that the stool submission process was less valuable because diarrhoea was often self limiting. One GP stated samples were sent too often (Table 2).

### Most common reasons for sending a sample

Overall the most common reason (17; 85%) for submitting a stool specimen was long duration of illness, followed by recent travel (8; 40%) (Table 3). When specifically asked to clarify the term “prolonged diarrhoea” there was considerable variation in opinion from 5 days to over three weeks; five stated over five days, nine one week and three over two weeks; there was no difference in this definition between GPs from lower, average and higher submitting surgeries. About a quarter mentioned blood in the stool, patient unwell, and to exclude infection as other reasons they had sent specimens. However only two GPs said that they would specifically ask the patient whether there was blood and/or mucus in the stool. Other reasons mentioned by one or two GPs included recent antibiotics, underlying illness, systemic symptoms, patient anxiety and illness of other family members (Table 1).

### Public health issues GPs’ discussed with patients

About half of the interview participants reported that they would specifically question patients with diarrhoea about recent antibiotic use, the risk of food poisoning, a patient’s occupation or other public health issues. However nine GPs reported that they would NOT specifically ask a patient about recent antibiotic use, as they indicated that this information would be gleaned from the patient’s notes. Ten GPs reported that they asked their patients about potential food poisoning and specifically asked about the foods consumed, whilst two GPs stated that they would wait for the patient to volunteer information about what they have eaten. Although more than half of GPs stated that if the patient’s occupation related to food handling they would be concerned (11), only 25% of GPs would specifically ask a patient about their occupation during a consultation. If the patient was a food handler, five GPs volunteered that they would advise the patient to take time off work and six GPs would send a sample sooner in food handlers (Table 4).

Nine GPs (45%) would ask whether the patient’s friends, family or colleagues were experiencing similar symptoms and of these, the majority reported that they would investigate possible sources of food poisoning (7) and only one GP volunteered that they would specifically enquire about contact with children. Three of nine participants questioned about obtaining a history of farm visits to rule out *Escherichia coli* reported that they would investigate this specific risk factor.

### When GPs send samples requesting Ova, cysts and parasites (OCP)

When questioned about when they would consider OCPs, 65% (13) of GPs stated they would send a sample for OCP if the patient had recently returned from travel abroad. Four GPs incorrectly assumed that as they requested “M, C & S” (microscopy, culture and susceptibilities) on stool samples this also included microscopy for ova, cysts and parasites. Other circumstances for suspected OCP included prolonged diarrhoea (2), patient unwell with weight loss (2), suspected giardia (2), blood and mucus in the stool (1), watery diarrhoea (1) and patient’s occupation i.e. working in sewers (1) (Table 5).

### Process of obtaining stool specimens from patients with diarrhoea

GPs were asked about the process they followed once they had decided to request a stool specimen from the patient. Half of the GPs (10) stated that when giving advice they “*Print off the form, give them the pot, tell them what to do and tell them to bring it back”* or a variation on these four steps. Five GPs discussed in detail tips to collect the sample e.g. using kitchen paper, ice cream tubs or cling film on the toilet. The varied advice given by GPs indicated that the majority were not clear themselves about exactly what instructions should be given, how much stool should be collected, or the exact storage requirements. Although GPs listed many barriers associated with patient stool specimen collection (unwillingness or difficulty with process (9), unable to return to surgery (5), elderly or child (6), physical or learning disability (3), the majority of GPs interviewed (9) and in the discussion group reported that most patients still provided a sample “*they find a way*”. No GPs reported giving patients any written collection advice. When shown the HPA patient information leaflet on how to collect a specimen (Additional file 2: Appendix 2), nine GPs agreed that they would give a printed version to their patients to help explain how to collect the specimen (Table 6).

The majority of the GPs reported submitting specimen requests electronically (12), the remaining still used paper request forms (8). Clinical information given on the request forms, tallied with the most common reasons for submission above. All participants were asked their opinion on why many clinicians simply write just ‘diarrhoea’ on the request form. Most felt this was probably due to time pressures (9), or laziness (2), ease (2), not realising that additional information could lead to further testing (3), support or locum staff (2). For about half of GPs this quote summarises their approach to their submission of stool specimens: “*I don’t have much sort of sophistication in terms of exactly what I’m asking for I’m afraid.”* To improve the clinical information provided GPs requested more advice on the information needed (3), and more detailed or tick boxes on the form (8) (Table 7).

### Interpretation and value of microbiology stool report results

Seven GPs interviewed realised that a negative result did not exclude all causes of infectious diarrhoea but the majority of interviewees took the negative report at face value and understood a negative result as meaning that no pathogenic organisms were present (Table 8).

Most GPs (13) stated the microbiology laboratory reports were returned to them personally and felt reports were useful and valuable; 80% said that they influenced their management, mainly because reports helped identify if specific antibiotic treatment was needed (9 participants), helped to make a definitive diagnosis (4), helped to provide advice for patients (3) and excluded serious infection (2). However over half of the GPs (11) noted that they would treat a patient before receiving a microbiology laboratory report.

Two-thirds of GPs suggested improvements to the reports; including more detailed advice on management and the meaning of the result (including negatives) especially if the infection was unusual. One GP requested a faster service.

### Sources of advice for GPs on when to submit stool samples

Most GPs interviewed (17) reported that they would contact their microbiology laboratory or use local hospital guidance (2) if they were unsure whether to send a stool specimen. Three GPs claimed they never accessed any source of guidance, as it was not easy to access clear information (2); they relied on their own experience (Table 9).

### Usefulness of stool specimen guidance to GPs in patients with diarrhoea

Most GPs stated written guidance would be useful (12) and felt that it would be best placed on the surgery intranet (7) for ease of printing, or attached to the laboratory report (6). Only three thought paper copies were useful. Eighty percent of GPs were not aware of the HPA guidance (16), and only one GP had used it [7]. Similarly, 80% of GPs were not aware of the CKS guidelines, yet half had heard of Prodigy – the previous name for the CKS guidance. When given the opportunity to review the HPA guidance [7], 35% of interviewed GPs specifically stated they would use the guidance now they had seen it. GPs commented that the advice was clear (7), helpful (6) and easily accessible (1). Forty five percent of GPs (9) interviewed felt that the information on the second page of the HPA guidance [7] (Additional file 2: Appendix B) about how to collect a specimen was useful and could be used as an advice leaflet for patients. Twelve GPs suggested that the guidance could be improved by reducing the amount of information on each page. Nearly half of the interviewed GPs would not utilise the reference section (7) (Table 10).

### Discussion group guidance results

None of the 24 GPs at the discussion group had previously seen the HPA guidance and all were unaware that it was freely available on the HPA internet pages. Group participants suggested that locating the guidance on the surgeries intranet (n = 2) or providing links to it, or “pop ups” when GPs electronically requested microbiology for stool samples (n = 7) would be useful. All the GPs in the discussion group agreed that the guidance on when to send a specimen and the history required was useful and none voiced that any additional information was required. All participants agreed that they liked the patient information leaflet on how to collect a specimen and reported that they would give a printed version to their patients to help explain how to collect it. One GP cautioned about the length of the guidance “…*it’s probably too much though; you wouldn’t want to read it all”.* Two GPs suggested that the references and grading provided were not useful to them as they trusted the guidance given by the HPA and didn’t need proof within the guidance “*if this is what you want us to do then we’ll do it and we’ll trust you’ve got your information from a reliable source”.*

Like the interviewees the GPs in the discussion group took a negative stool report at face value, and were surprised that the laboratory would only screen for the organisms listed under the “Pathogens routinely looked for” section of the guidance and that additional clinical details would be required to prompt the laboratory to screen for the organisms listed under the “Other enteropathogens looked for depending on history” section. The discussion group participants were surprised when the moderator confirmed that the final report only lists those few organisms routinely looked for: “*Does that mean that if you (the laboratory) send out the form and it says that so and so has not been found, that that is all they’ve looked for?”*

## Discussion

### Main findings

The majority of interview participants reported that stool specimen microbiology was a useful and positive process when indicated, based on the individual patient’s symptoms. They reported that stool submission should not be routine, as the majority of patients with IID (as indicated in the HPA guidance) do not usually require any specific antibiotic treatment and that IID is usually self-limiting. The most common reasons for submitting a stool specimen were for longer duration of illness and recent travel. Only two GPs specifically asked patients whether there was blood and/or mucus in their stool; only half asked about recent antibiotic use, or potential food poisoning, and few would specifically ask a patient about their occupation. The most common reason for requesting microscopy for ova, cysts and parasites in stool specimens was if the patient had recently returned from travel abroad. Although GPs listed many barriers associated with patient stool specimen collection, few gave patients advice on how to collect them and only one GP in this study had used the HPA stool guidance or the HPA patient stool collection sheet within the guidance, however the majority reported that patients seemed to manage to provide a specimen when requested. The majority of the GPs submitted specimen requests electronically and would value links to this or other guidance on the intranet readily available when they submitted specimens. Many suggested that a better tick box system on request forms would help them to give more relevant clinical details to target the investigations undertaken. Many GPs thought that a negative stool report indicated that no pathogenic organisms were isolated, and they were all surprised that the list of pathogens not isolated was all that was looked for.

Over half of the GPs reported that if they suspected campylobacter infection they would give antibiotic treatment before receiving laboratory confirmation. This is sensible as the severe abdominal pain and food history may give strong indication of the cause and antibiotic treatment needs to be given early to have a significant effect on duration of illness. However, undoubtedly many patients will receive unnecessary treatment and this is an area where a near patient molecular test would be extremely helpful in directing specific and timely treatment.

### Strengths and weaknesses

When data collection ceased, the themes themselves were being replicated indicating a level of completeness. And as we recruited GPs served by three different laboratories with a range of submission behaviour and 80% of those selected agreed to participate, we consider that we have probably identified a representative range of opinions from GPs in England about stool specimen collection. There is no evidence that reimbursing clinicians for their time to participate in the interviews led to selection bias, as we had such a high agreement to participate and a range of opinions about stool submission. We used qualitative methods rather than quantitative so that we could probe participants about their answers and fully understand them, thus the information we obtained was much richer and broader than we could have gained in a questionnaire which usually ask closed questions with a restricted number of possible answers. Qualitative research does not aim to determine the statistical significance of a particular opinion or behaviour, rather it aims to gather the range of opinions held by a particular group and give some indication of how commonly held the opinions or behaviours are. The data generated can then be used to inform questionnaire development – indeed we now intend to undertake a questionnaire survey of GPs from the three areas which will aim to confirm the most important findings in a much larger sample.

We used content analysis which is a quantitative framework that is most often used with written text, for summarizing any type of content using counting. This enables a more objective evaluation than comparing content based on the impressions of the researcher, and is ideal when the purpose is simply to understand the range of behaviour and opinion within a defined area. Bias introduced in using preset codes for the identification of relevant themes was reduced through developing additional codes if there was sufficient evidence to do so. The results of content analysis are numbers and percentages. Though it may seem crude and simplistic to make such statements, the counting serves to remove much of the subjectivity and simplifies the detection of trends. Identifying the most frequent responses is in line with the principles of content analysis and provides an indication of the most salient points. Content analysis is particularly useful in qualitative audit when questions have been based on a set of recommendations in guidance as was the case in this study.

Care was taken to minimise the interviewer influencing participants’ responses by choosing interviewers trained in qualitative research who were independent of the HPA, and ensuring that GPs were aware that their responses would be anonymised before being shared with other members of the project team who were HPA employees. The discussion group was facilitated by a biomedical scientist (BMS) who did not develop the guidance but had a good understanding of it. This allowed the BMS to discuss the clinicians’ understanding of the guidance and microbiology reports. It is possible that this may have introduced some acquiescence bias into the discussion group. The discussion group was held as part of an educational session and therefore may have included a biased sample of GPs with a particular interest in gastrointestinal infections; however none of the GPs had previously seen the stool guidance and were therefore able to give a fresh view of them.

### Other work in this area

Tam *et al.* estimated that in 2009 there were up to 17 million sporadic, community cases of IID and 1 million GP consultations in the UK [6]. The ratio of GP consultations to national surveillance figures in Tam’s study is much higher for viruses (norovirus ratio12.7; adenovirus ratio 15.3) than for bacteria (*Campylobacter* 1.3 and *Salmonella* 1.4). The reasons for these differences are complex. First the IID2 Study included sporadic cases, not cases from outbreaks. Virology is not routinely requested or performed for sporadic cases presenting to GPs. Secondly, as we found in our study, GPs were probably more likely to submit samples from patients with diarrhoea lasting for more than one week or after travel, both of which are more likely in bacterial cases of diarrhoea. Finally, since enteric viruses may present with predominantly vomiting rather than diarrhoea, and vomit samples are less likely to be sent off for analysis. Previous studies have shown that individuals with a greater disease severity and recent foreign travel are also most likely to consult, which will tend to reinforce GPs behaviour to sample in this group [10]. In a recent Japanese telephone survey 10% of patients who attended their GP with diarrhoea submitted a stool sample [11]. In an American survey in 2000 undertaken by the Centers for Disease Control and Prevention (CDC) 19% of those seeking medical care provided a stool sample; bloody diarrhoea (odds ratio (OR) 3.35) and diarrhoea duration over three days (OR 3.81) were the most important factors associated with submission [12]. Scallan *et al.* concluded that cases of acute diarrheal illness ascertained through laboratory-based public health surveillance are likely to differ systematically from unreported cases and likely over-represent those with bloody diarrhoea and longer diarrhoea duration [12]; we concur.

The Public Health (Control of Disease) Act 1984 requires cases of suspected food poisoning to be reported by any medical practitioner to their local Health Protection Unit [3] and since the early 1980s food poisoning notifications have steadily risen [4]. However, we found that the majority of GPs did not specifically ask patients about possible sources of infection or public health risks, and mainly waited for patients to volunteer such information. We have found no other qualitative research in this area, but a small audit in a single British GP surgery, found that in 47 of 59 cases of suspected infectious gastroenteritis or diarrhoea a potentially significant zoonotic source of infection was missed; this included keeping exotic or domestic pets and visits to pet shops, farms, zoos and other wildlife centres [13]. GPs in our study suggested that a history prompt for risks for acquiring IID when submitting specimens may allow them to provide more relevant history for the lab and remind them about possible public health risks and sources of infection.

## Conclusions

### Implications of this audit for policy makers, commissioners and microbiology laboratories

GPs value stool culture and we consider that it should continue to be provided. All GPs reported that the patient instruction leaflet on how to take a stool specimen was very useful, and this sort of information should be made more widely available with stool collection kits, as very few of the GPs that participated were aware of it or used it. The national stool guidance on when to take stool specimens needs greater promotion to GPs; and should be made available through creating weblinks or “pop ups” on the request form and via links within antibiotic guidance, which the majority of GPs do use. Detailed referencing of the guidance is not necessary routinely, but should be accessible when needed via the weblinks. The use of drop down tick boxes or prompts on electronic request forms would enable GPs to give more relevant clinical details to inform laboratories, should be trialled. As GPs do not all understand the meaning of a negative microbiology stool report, and would value more interpretation and treatment advice on a positive report, the labs should routinely give more advice and explanation on all reports; this could be supplemented with links to web based guidance. Continuing professional development should include this area of laboratory medicine, which is often overlooked.

As GPs only send stool specimens in a subset of patients presenting with gastro-intestinal symptoms, mainly post travel or after prolonged or severe symptoms, surveillance of IID will tend to lead to a biased reporting sample with under reporting of all cases of mild IID and greater reporting of bacterial infections compared to viral infections that are usually short lived. Other methods of surveillance will be needed to monitor sporadic cases of viral IID. The development and accessibility of near patient molecular tests would help to direct the acute management of patients with diarrhoea in the GP surgery.

### Implications of this audit to clinicians

So that diagnosis of severe cases of IID and identification of those with public health issues are not delayed, GPs need to have a more systematic approach to deciding when to submit a stool sample. This should include eliciting patient signs and symptoms which indicate greater risks of severe infection (such as bloody diarrhoea and recent history of antibiotics), and enquiring about specific points in the patient history to identify the source of infection and any important public health issues, including food related illness, possible outbreaks, occupation and farm visits. Enquiring about the possibility of food poisoning will give GPs the information they need to undertake the mandatory reporting of suspected food poisoning to their local Health Protection Unit; GPs should also remember that this should include mild cases. Knowledge of the important areas to cover could be attained by accessing the HPA [7] or CKS [8] guidance. GPs should give patients more information about how to collect a stool sample.

## Competing interests

The authors declare that they have no competing interests.

## Authors’ contributions

CM suggested and led the audit, wrote the protocol and drafted questions for the interview schedule, participated in the analysis and wrote the paper. GL assisted with the protocol, helped devise the questions for the interview schedule, undertook the discussion group, participated in the analysis; gathered and analysed the laboratory data, wrote the initial draft of the paper and commented on the final paper. KN and PJ – commented on the protocol, helped devise the interview schedule, undertook GP interviews, analysed the interview data and commented on the paper. HY, KK, SO and ME – commented on the protocol, assisted with the questions for the interview schedule and commented on the paper. All authors read and approved the final manuscript.

## Pre-publication history

The pre-publication history for this paper can be accessed here:

http://www.biomedcentral.com/1471-2296/13/77/prepub

## Supplementary Material

Additional file 1 ** Appendix A.** GP audit of stool specimen submission - interview and discussion group schedules. Click here for file

Additional file 2 ** Appendix B.** HPA infectious diarrhoea guidance. Click here for file

Additional file 3 ** Appendix C.** Definitions of codes and definitions under objectives. Click here for file
