# Supplementary material for: Stool submission by general practitioners in SW England - when, why and how? A qualitative study
Source: BMC Fam Pract. 2012 Aug 8;13:77. doi: 10.1186/1471-2296-13-77 (PMC3481435; doi:10.1186/1471-2296-13-77)
Supplement: Additional file 1 — Appendix A. GP audit of stool specimen submission - interview and discussion group schedules. [file 1471-2296-13-77-S1.docx]

**Appendix A: GP audit of stool specimen submission - interview and discussion group schedules**

*Highlighted sections were also asked of discussion group participants as part of the process of going through the guidance section by section*

**Complete pre-interview demographic survey prior to interview**

*Re-iterate to participant that we are independent of the HPA and that their responses will be anonymised*

*Advise participant that the first set of questions is about stool sampling*

1. Your patients probably have different ideas about what diarrhoea means. How would you describe it to them?

*This is to identify whether GPs have a consistent and accurate understanding of what is meant by diarrhoea (B)*

1. What are your thoughts on the value of sending stool samples for microbiology?

*Make sure get positive and negative opinions about value. This is to identify whether GPs have positive attitudes towards stool sampling. To investigate whether GPs think submitting a stool sample to the lab is worthwhile. (A)*

1. Roughly how often do **you** send stool samples for microbiology? (Try to gain an estimate of how many per month)

*This is to identify and compare self-reported behaviour across the sample (D)*

1. Roughly what percentage of patients with diarrhoea do you send specimens for?

*This is to identify and compare self-reported behaviour across the sample (D)*

1. Do you submit diarrhoea specimens with electronic forms?

*This is asked because if electronic, then there is an opportunity to place guidance alongside.*

1. Compared to other clinicians in your practice do you think **you** have a high or low rate of submissions? (Ask participant to explain why they think they fall into this category; Do they feel they could/should be sending more or less samples – why?)

*This is to identify GPs perceptions about whether they compare favourably to their colleagues and to allow us to make a supposition about whether the participant is typical of their practice (for which we have an actual submission rate) (D)*

1. What sort of information do you put in the clinical details box when submitting specimens for microbiology? Identify whether the participant routinely just writes ‘diarrhoea’. Why do others just put diarrhoea? What would help improve the reporting of clinical details by GPs on submissions?

*This is to identify whether GPs are providing sufficient information to the microbiology labs to enable them to carry out appropriate testing (F)*

1. What are your most common reasons for sending a stool sample for microbiology? Prompt: What from the history would prompt you to request a stool specimen? Was it because of clinical symptoms, number of times patient consulted, because patient in particular risk group, risk to community etc. If only send in chronic diarrhoea (i.e. lasting more than 14 days) – explore reasons why don’t send in acute cases.

*This is to identify most common reasons for submitting samples and whether these are in line with the guidance (C) (F)*

1. What from the history would prompt you to think the illness is **serious** and requires a definitive diagnosis? Prompt for serious to the individual (i.e. elderly or very young, pregnant, long-term weight loss, duration of illness, recurrent diarrhoea and so on), and serious to the wider community at risk i.e. see if mention: diarrhoea in food handlers, health professionals, children attending nurseries, elderly in care homes; whether there has been a farm visit; where there is evidence of outbreaks in family/community (as discussed in HPA guidance).

*This is to identify GPs understanding about what clinical details would prompt them to think that a particular case is particularly serious and in need of escalation i.e. informing HPA, potentially screening for other family members (C) (F)*

1. In patients who present with diarrhoea, do you usually ask if this could be food related? Do you ask about any other public health risks? Prompts: do they ask what eaten, where, are they a food handler, others members of public at risk (NB could be patient presenting with chronic or acute diarrhoea).

*This is to identify whether GPs routinely consider the possibility that diarrhoea is food-related and what they have understanding is about the implications of this. Although similar to Q7 this is examining food related infections specifically i.e. those often caused by poor hygiene in food handlers passed through preparing salads etc. Again implications for public health e.g. elderly in care homes and children in nurseries (C) (F)*

1. Do you ask about antibiotic use?

*This is asked because some antibiotics cause diarrhoea*

1. When would you consider asking for ova, cysts and parasites? Prompt for specific rules of thumb (e.g. been abroad recently and where, had contact with animals, potential contact with contaminated food or water sources, child attending nursery/school etc) and what information is collected to make decision. What information do you put on the form?

*This is to identify compliance with the guidance. Samples are normally sent for bacterial investigation, as the majority of infections in this country are bacterial in aetiology. However, for example if patients have been on holiday to a certain country where parasites are endemic, the GP would need to communicate this with the lab to ensure that the lab screens the patient’s sample for parasites (C) (F)*

1. If a stool sample is needed, what do you do next? Prompt with: pots, request form, instructions for taking specimen, information leaflets? And where are these kept?
2. ‘Can you identify any particular barriers to getting a patient to provide a stool sample?’

*These two questions are to identify what GPs gather in preparation for taking a stool sample and how accessible these things are (e.g. whether stool pot and lab report forms are readily available in GPs room or at reception). It also includes identification of other barriers to behaviour such as patient attitudes (E)*

What advice do you give patients about collecting and returning samples?

Prompts:

‘Do you advise on

How to collect a sample?

The amount of stool to collect?

If you suspect ova, cysts and parasites, how many samples to you advise they provide and over what time period?

What patients should do once the sample has been collected?

How soon they should return the sample to the practice?

What do you advise the patient to do if their diarrhoea has resolved by the time they pass their next stool?

*This is to identify whether GPs are giving advice in accordance with the guidance (F)*

Is there anything else you want to say about when you request stool specimens and how they are collected?

*Advise the participant that this second set of questions is about stool microbiology reports*

1. Are microbiology reports from specimens that you have requested always returned to you personally? Probe: if not who receives them, how is patient followed-up i.e. contacted about results and possible treatment

*This is to identify the continuity of care (H)*

1. What are your thoughts on the value of stool microbiology reports? (Explore reasons – cover positive and negative reports; they way results are presented).
2. To what extent do the reports influence your management? (i.e. do you do anything differently as a result?).

*These two questions are to identify GPs perceptions of the value of microbiology reports (G) (H)*

1. How would you describe the quality of information provided in the microbiology reports? NB Cover positive and negative reports. Prompts: Is the information always clear? Is enough information provided to enable them to decide on clinical management?

*This is to identify whether GPs perceptions on the quality of information provided (G) (H)*

1. What do you understand by a negative report?

*This is to identify whether GPs understand that negative reports do not mean that all pathogens are excluded (F) (G) (H)*

1. What are your thoughts on how likely you are to receive false-negative reports? Explore including how affects clinical management of patients.

*This is to further identify GPs perceptions of the value of reports (G) (H)*

1. In what way could the microbiology report be improved? Explore: content, layout, comments provided by the lab.

*This is to identify GPs perceptions of how the reports could be improved (G)*

1. Under what circumstances would you prescribe antibiotics for diarrhoea? (Is it before or after getting lab results? Explore how this is of influence if at all)

*This is to identify the extent to which GPs may be inappropriately prescribing antibiotics for diarrhoea and whether the information provided in the microbiology report influences this*

*Advise participant that this third set of questions is about guidance.*

1. If you were uncertain about whether to send a stool specimen for microbiology, where or to whom would you look for guidance?
2. (If training practice) Where do you advise trainees to go for further advice?

*These two questions are to identify current sources of information/support and use of guidance (I) (J)*

1. Would written guidance on when and how to take specimens, and how to treat diarrhoea, be useful to you? If yes, where is this best placed? I.e. attached to report or electronic copy?

*This is to identify awareness of guidance and value of guidance (I) (J)*

1. Do you know the Health Protection Agency provides guidance for GPs on sending specimens for microbiology? Explore use and immediate thoughts on it.

*This is to identify awareness of the guidance (J)*

1. Are you aware of the CKS (Clinical Knowledge Summary) guidance? Prompt: Explore use and immediate thoughts on it. NB older doctors may refer to/remember it as ‘Prodigy’ guidance.

*This is to identify awareness of the guidance (J)*

1. What are the advantages/disadvantages of using guidance to support the clinical management of diarrhoea? (explore views on clinical guidance in general)

*This is to identify GPs perceptions of the value of guidance (I)*

*Comment on content of HPA guidance*

*Ask the participant to open the envelope or email containing the HPA guidance and ask them:*

1. If they have seen this guidance before
2. If they have used this guidance before
3. What their immediate thoughts are on its contents (how useful) and layout

*Inform participant that we will send a copy of the transcript for them to review for completeness/accuracy.*

Discussion group participants were shown each section of the HPA infectious diarrhoea guidance (Appendix B) and asked to comment on the content and layout.
