# Supplementary material for: Stool submission by general practitioners in SW England - when, why and how? A qualitative study
Source: BMC Fam Pract. 2012 Aug 8;13:77. doi: 10.1186/1471-2296-13-77 (PMC3481435; doi:10.1186/1471-2296-13-77)
Supplement: Additional file 2 — Appendix B. HPA infectious diarrhoea guidance. [file 1471-2296-13-77-S2.docx]

**Appendix B: HPA infectious diarrhoea guidance**

| 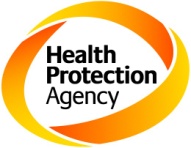 Infectious DiarrhoeaThe Role of Microbiological Examination of FaecesQuick Reference Guide for Primary Care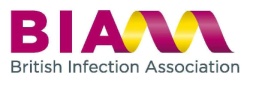 For consultation and local adaptation | | | | | | | | | | | | | | |
| --- | --- | --- | --- | --- | --- | --- | --- | --- | --- | --- | --- | --- | --- | --- |
| B- | - **Definition of acute diarrhoea: 3 or more episodes a day, <14d and sample takes shape of pot.^1,2,3^** - **About 20% of the population develop infectious intestinal disease (IID) per year. ^4^** - **Most infectious diarrhoea is a self-limited, usually viral illness. Nearly half last less than one day.^2,5^** - **If the diarrhoea has stopped, culture is rarely indicated, as recovery of the pathogen is unlikely.** - **Infectious diarrhoea should be considered in parallel with other causes of diarrhoea.**   **Pathogens routinely looked for (cases per 100,000 population 2005):** | | | | | | | | | | | | | |
|  | - Campylobacter (87 cases) - Cryptosporidium (9 cases) | | | | | | - *Escherichia coli* *0157:H7* (2 cases) - Salmonella (21 cases) | | | | | - Shigella (2 cases) | | |
|  | **Other enteropathogens looked for (depending on history):** | | | | | | | | | | | | | |
|  | - Noroviruses - Rotavirus - *Giardia lamblia* | | - *Entamoeba histolytica* - *C. difficile* | | | | - Vibrio - *Yersinia enterocolitica* - Cyclospora | | | | - Toxin-producing *Clostridium perfringens* - *Bacillus cereus* - *Staphylococcus aureus* toxin | | | |
| B- | **WHEN TO SEND A FAECAL SPECIMEN^2,3^** | | | | | | | |  | **WHEN ADVISED BY HEALTH PROTECTION UNIT^2,8,9^** | | | |  |
|  | - Patient systemically unwell; needs hospital admission and/or antibiotics. - Blood or pus in stool. - Acute painful, or bloody diarrhoea in previously healthy children to exclude *E. coli* O157 infection^6^ - Post antibiotics and hospitalisation (*C. difficile*). - Diarrhoea after foreign travel; you should request ova, cysts and parasites (OCP). - Persistent diarrhoea when Giardia is suspected.^7^ - For reassurance, as diagnosis of infection may exclude other pathologies. | | | | | | | | - Suspected public health hazard:   e.g. diarrhoea in food handlers, healthcare workers, children after farm visits (*E. coli O157*), or at nurseries, elderly residents in care homes or other high-risk situations.   - Outbreaks of diarrhoea in family, community, etc when isolating the organism may help pinpoint outbreak source. - Contacts of patients with certain organisms e.g. *E. coli* 0157, where there may be serious clinical sequelae. | | | | | |
|  | **WHAT TO SEND (see next page for how to collect)** | | | | | | | | | | | | | |
| **C**  **B** | - Single specimen (minimum needed for routine investigation only: 1 ml - size of pea). - If recurrent, send 3 specimens (5 ml each) 2-3 days apart, as OCP are shed intermittently.^10^ | | | | | | | | | | | | | |
|  | **HISTORY THAT SHOULD BE INCLUDED ON FORM TO HELP DETERMINE DIAGNOSTIC METHODS** | | | | | | | | | | | | | |
| **C** | - **Thorough clinical evaluation of a patient is needed to guide laboratory testing and therapy.** - **Please state if submitted at request of HPU, CCDC or EHO.** | | | | | | | | | | | | | |
| **B** | **Clinical features:**   - Systemic illness, fever, bloody stool - Symptoms; duration, recurrent, chronic - Severe abdominal pain (Campylobacter) - Immunosuppression | | | | | | | | **Epidemiological setting:**   - Food intake e.g. barbecue; restaurant; eggs; chicken; shellfish - Recent foreign travel and to which country - Recent antibiotic, PPI or hospitalisation (*C. difficile*)^11,12^ - Family or nursing home; (Norovirus) - Exposure to untreated water (protozoa) or animals - Contact with other affected individuals or outbreak | | | | | |
|  | **INTERPRETING THE LABORATORY REPORT** | | | | | | | | | | | | | |
| **B** | - A pathogen is found in only 2 – 5% of specimens submitted.^3^ - A negative report does not mean all pathogens are excluded; the pathogens sought will usually be listed.   e.g. There are no routine methods for detecting enterotoxigenic *E. coli*, the commonest cause of traveller’s diarrhoea. | | | | | | | | | | | | | |
|  | **TREATMENT FOLLOWING REPORT** | | | | | | | | | | | | | |
| **B**  **A**  **C** | - Most patients in whom pathogens are detected will NOT require specific treatment unless systemically unwell or treatment is advised by a microbiologist or consultant in communicable disease control. - Urgently refer all previously healthy children with acute painful, bloody diarrhoea or confirmed *E. coli* O157. - *C. difficile:* Stop unnecessary antibiotics and/or PPIs to re-establish normal flora. Prescribe metronidazole 400 or 500 mg oral tds. 70% of patients respond after 5 days; 94% in 14 days. Monitor >85 year olds as mortality double.^13,14^ If severe (characterised by T >38.5; WCC >15; rising creatinine or signs/symptoms of severe colitis) or 3^rd^ episode, prescribe vancomycin 125mg oral qds for 10-14 days. - Campylobacter: Antibiotic therapy has little effect on duration of symptoms unless given very early in illness course.^15^ - *G.* *lamblia* and *E. histolytica* should be treated. ^7^ - Unless symptoms persist, Blastocystis & *Dientamoeba fragilis* do not usually require treatment in otherwise healthy.^16,17^ | | | | | | | | | | | | | |
|  | **WHEN TO SEND A REPEAT SPECIMEN** | | | | | | | | | | | | | |
|  | - Usually unnecessary unless advised by a microbiologist or consultant in communicable disease control. | | | | | | | | | | | | | |
|  | **KEY** | **A** | | **B** | **C** | **D** | | **Indicates grade of recommendation** | | | | |  | |

**Collecting a stool specimen for microbiological examination**

1. DO NOT mix urine with the stool sample. If you need to pass water, do so first.
2. Place a wide mouth container (potty, empty plastic food container e.g. 1 litre ice cream carton) in the bowl, or put clean newspaper or plastic wrap over the toilet seat opening (this prevents the faecal/stool specimen from falling into the toilet bowl. (Collection container does not have to be sterile, but must be clean).
3. Pass stool onto the potty, plastic container, newspaper or plastic wrap.
4. Using the spoon built into the lid of the collection tube (or the wooden sticks, if supplied), place small scoopfuls of stool from areas which appear bloody, slimy or watery into the tube. DO NOT OVERFILL. Try not to spill stool on the outside of the tube.
5. Replace the collection tube lid and screw on tightly.
6. Dispose of remaining stool in your potty, plastic container or newspaper down the toilet. Clean potty with hot soapy water. Wrap plastic container, newspaper or plastic wrap in newspaper and dispose of in normal refuse in a plastic bag.
7. Label the collection tube with your name, date of birth and the date of collection
8. Place the container in the plastic bag attached to the specimen request form.
9. Wash your hands thoroughly in hot running water with soap.
10. Deliver to the surgery/laboratory as soon as possible.
11. If specimen cannot be delivered immediately, refrigerate in surgery fridge until delivery.


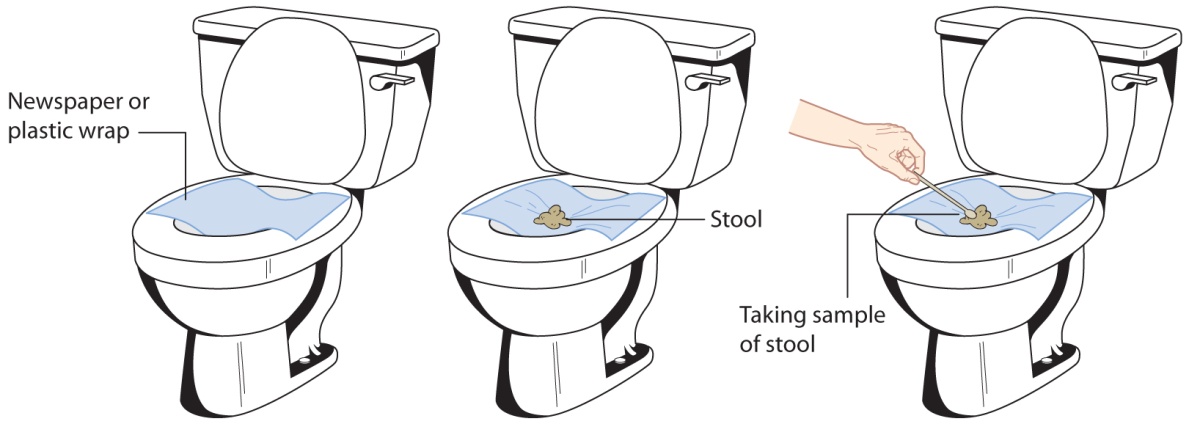


**Grading of guidance recommendations**

| **Study Design** | **Recommendation Grade** |
| --- | --- |
| Good recent systematic review of studies | A+ |
| One or more rigorous studies, not combined | A- |
| One or more prospective studies | B+ |
| One or more retrospective studies | B- |
| Formal combination of expert opinion | C |
| Information opinion, other information | D |

**This guidance was updated in July 2010 following the Griffin Report into the investigation of an outbreak of Escherichia coli O157 led to severe illness in a number of visitors to Godstone Farm in Surrey.^7^**

**References**

1. Al-Abri SS, Beeching NJ, Nye FJ. Traveller’s diarrhoea. *Lancet* *Infect Dis* 2005;**5**:349-60.
2. Thielman NM, Guerrant RL. Acute infectious diarrhoea. *N Eng J Med* 2004;**350(1):**38-47.
3. Geurrant RK, Gilder TV, Steiner TS, Thielman NM, Slutsker L, Tauxe RV, Hennessy T, Griffin PM, DuPont H, Sack RB, Tarr P. Neill M, Nachamkin I, Reller LB, Osterholm MT, Bennish ML, Pickering LK. Practice guidelines for the management of infectious diarrhoea. *Clin Infect Dis* 2001;**32**:331-51.
4. Wheeler JG, Sethi D, Cowden JM, Wall PG, Rodrigues LC, Tompkins DS, Hudson MJ, Roderick PJ on behalf of the Infectious Intestinal Disease Study Executive. Study of infectious intestinal disease in England: rates in the community, presenting to general practice, and reported to national surveillance. *BMJ* 1999;**318**:1046-50.
5. Herikstad H, Yang S, Van Gilder TJ, Vugia D, Hadler J, Blake P, Deneed V, Shiferaw B, Angulo FJ and The Foodnet Working Group*.*  A population-based estimate of the burden of diarrhoeal illness in the United States: FoodNet, 1996-7. *Epidemiol* Infect 2002;**129**:9-17.
6. The Griffin Report**.** Review of the major outbreak of *E. coli* O157 in Surrey, 2009

An evaluation of the outbreak and its management, with a consideration of the regulatory framework and control of risks relating to open farms. 2010 [www.griffininvestigation.org.uk](http://www.griffininvestigation.org.uk) . Accessed July 23^rd^ 2010.

1. Katz DE, Taylor DN. Parasitic infections of the gastrointestinal tract. *Gastroenterology Clinics of North America* 2001;**30**:797-815.
2. Working Group of the former PHLS Advisory Committee on Gastrointestinal Infections. Preventing person-to-person spread following gastrointestinal infections: guidelines for public health physicians and environmental health officers. *Commun Dis Public Health* 2004;**7(4)**:362-84.
3. Health Protection Agency. Algorithm for diagnosis, investigation and management of suspected cases of *E.coli*/Vero-cytotoxin producing *Escherichia coli* (VTEC). <http://www.hpa.org.uk/infections/topics_az/ecoli/O157/Algorithm_VTEC_3.pdf> . Accessed 9^th^ November 2007.
4. Van Gool T, Weijts R, Lommerse E, Mank TG. Triple faeces test: an effective tool for detection of intestinal parasites in routine clinical practice. *Eur J Clin Microbiol Infect Dis* 2003;**22(5)**:284-90.
5. Dial S, Delaney JAC, Barkun AN, Suissa S. Use of gastric acid-suppressive agents and the risk of community-acquired *Clostridium difficile*-associated disease. *JAMA* 2005;**294(23)**:2989-95.
6. Forward LJ, Tompkins DS, Breett MM. Detection of *Clostridium difficile* cytotoxin and *Clostridium perfringens* enterotoxin in cases of diarrhea in the community. *J Med Microbiol* 2003;**52**:753-57.
7. Health Protection Agency. *Clostridium difficile* Robotyping Network (CDRN) for England and Northern Ireland. 2009/10 Report, page 26: Available at <http://www.hpa.org.uk/Publications/InfectiousDiseases/InfectionControl/1102cdiffcdrnCDRNannualreportEW/> Accessed 12^th^ July 2011. *This report shows a multivariate analysis of factors associated with mortality in a 24 month period 2008-2010. Age >60 vs <60 yrs had over 2.5-fold mortality; severe CDI had 5-fold mortality; ribotype 027 had 2-fold mortality.*
8. Miller M, Gravel D, Mulvey M, Taylor G, Boyd D, Simor A, Gardam M, McGeer A, Hutchinson J. Moore D, Kelly S. Health care-associated *Clostridium difficile* infection in Canada: patient age and infecting strain type are highly predictive of severe outcome and mortality. *Clin Infect Dis* 2010;50:194-201.

*This Canada-wide 2005 study collected data in 1008 patients with C. difficile. Patients 60-90 years were twice as likely to experience a severe outcome; this was increased if the infection was due to ribotype 027.*

1. Ternhag A, Asikainen T, Giesecke J, Ekdahl K. A meta-analysis on the effect of antibiotic treatment on duration of symptoms caused by infection with campylobacter species. *Clin Infect Dis* 2007;**44**:696-700.

*(Antibiotic treatment shortens duration of diarrhea by 1-3 days. Duration of symptoms was shorter [2.4 vs 4 days] if treated within 3 days of start of symptoms versus ≥ 3 days.)*

1. Tan KSW. Blastocystis in humans and animals: new insights using modern methodologies. *Vet Parasitol* 2004;**126**:121-44.
2. Lagacé-Weins PR, Van Caeseele PG, Koschik C. *Dientamoeba fragilis:* an emerging role in intestinal disease. *CMAJ* 2006;**175(5)**:468-9.
