# Supplementary material for: Stool submission by general practitioners in SW England - when, why and how? A qualitative study
Source: BMC Fam Pract. 2012 Aug 8;13:77. doi: 10.1186/1471-2296-13-77 (PMC3481435; doi:10.1186/1471-2296-13-77)
Supplement: Additional file 3 — Appendix C. Definitions of codes and definitions under objectives. [file 1471-2296-13-77-S3.docx]

**Appendix C: Definitions of Codes and definitions under objectives**

**Objective A: Do GPs value the process of stool specimen microbiology?**

1. **Positive attitude towards stool specimen microbiology**: Specifically positive comments about how the GP feels about stool specimen microbiology
2. **Negative attitude towards stool specimen microbiology:** Specifically negative comments about how the GP feels about stool specimen microbiology

**Objective B: On what basis do GPs make decisions about when to request a stool sample to send for microbiology?**

1. **Most common reasons for sending a sample**: If the GP goes into detail about prolonged diarrhoea and gives the length of time add this quote to code: ‘Prolonged diarrhoea’. If the GP goes into detail about a reason e.g. food related, risk group, blood or mucus also add this quote to the relevant code such as code: ‘Food related diagnosis’.
2. **Prolonged diarrhea**: The length of time the diarrhoea must remain for the GP to class it as prolonged and the length of time the GP wait’s to consider submitting a sample.
3. **Public health concerns:** Sub codes:

- **Diarrhoea in food handlers, healthcare workers**: GP specifically asks if the patient is in a food related job or not, they treat the patient differently on the basis of their job or not
- **Children after farm visits (E. coli O157), or at nurseries:** Does the GP investigate recent farm visits or not, does the GP ask any specific questions about children or are they concerned about children when making diagnosis
- **Elderly residents in care homes or other high-risk situations**: Does the GP ask if the patient works with elderly people or not, do they treat the patiently differently if they do or not, does the GP have any extra concerns if they do or not, does the GP treat the patiently differently if the patient in a resident of a care home
- **Outbreaks of diarrhoea in family, community**: When isolating the organism may help pinpoint outbreak source. Does the GP specifically investigate whether family/friends have symptoms? This can include quotes which fit into other codes e.g. ‘I ask if they have eaten anything dodgy and whether their friends ate it and felt the same’ would go here and in ‘investigating suspected food poisoning’ code.

1. **Patient history**: Does the GP investigate patient history or not? Factors from the patient history which influence their management of the patient: e.g. blood or pus in stool, patient systemically unwell, post antibiotics or post hospitalisation, diarrhoea after foreign travel, persistent diarrhoea, for reassurance, as diagnosis of infection may exclude other pathologies, medical history, food poisoning, quality of diarrhoea, length of diarrhoea
2. **Investigating possible food poisoning:** Does the GP specifically ask the patient if the diarrhoea could be food related or not, reasons for asking/not asking, if yes in what context e.g. bad food, foreign food from travel.
3. **Investigating antibiotic use:** Is the GP asking the patient specifically if they are taking/have recently taken antibiotics when investigating why diarrhoea could occur, include reasons for why/why they are not asking.
4. **Ova, cysts and parasites:** Does the GP consider asking about whether the diarrhoea could be caused by ova, cysts or parasites? Reasons why/why not if given, if details are given about what is submitted on the form in regards to ova, cysts and parasites then this quote should be under code: ‘Submitting a form for suspected ova, cysts and parasites’ as not every GP was asked.

**Objective C - What do GPs perceive as the barriers to collecting stool samples?**

1. **Barriers to collecting the sample**: What prevents the GP collecting a sample or interferes with the process e.g. patient attitudes, practicality, difficulty of collection amongst others.

**Objective D - Do the criteria that GPs and other primary care staff report using when submitting specimens concur with HPA and CKS guidance?**

1. **Process of collecting a stool sample:** What steps does the GP take after deciding to send a specimen, advice the GP gives to patients about how to collect a sample, advice about delivery of the specimen should be added only to code: ‘Advising the patient about delivery of the specimen’.
2. **Information provided at submission**: What information do the GPs put on the submission form e.g. how much detail do they go into, what aspects of patient history are specifically added to the submission form, level of detail.
3. **Electronic submission**: Are submissions made electronically or not, what systems (if electronic) do they currently use, any reasons given for why they submit electronically or not
4. **Suggested improvements to the submission processes**: Suggestion to improve the submission process, include comments the GP makes if no improvements are suggested and reasons why are given (e.g. the service is good already).

**Objective E - Do GPs value stool specimen reports?**

1. **Value of reports – negative:** Focuses on general impression of the report, the GP takes a specific view that returned microbiology reports are not useful or the GPs attitude is negative towards the usefulness of the report for diagnosis, treatment or quality.
2. **Value of reports – positive:** Focuses on general impression of the report, the GP takes a specific view that returned microbiology reports are useful/valuable or the GPs attitude is positive towards the usefulness of the report for diagnosis, treatment or quality.
3. **Negative returned reports**: What does the GP understand by a negative result in a returned report, include any comments about the influence to patient management.
4. **Understanding false negative results:** Does the GP understand the term false negative, chances of getting a false negative result and does this influence value placed on the report.

**Objective F - To what extent do stool specimen reports influence GPs clinical management of patients?**

1. **Influence of reports on clinical management**: The extent to which the report influences the clinical management of the patient including treatment, negatives and positive comments

**Objective G - What are GPs perceptions of the value of written guidance on submitting stool specimens?**

1. **Seeking guidance**: Sources the GP refers to for guidance before sending a specimen or enquiring about a returned report.
2. **Improvements to reports**: Suggested improvements to the microbiology reports, include comments if the GP suggests no improvements and gives reason why.
3. **Usefulness of written guidance**: How does the GP feel about using guidance, circumstances in which they would use guidance, accessibility issues of guidance, recommendations on where guidance would be best placed?
4. **Recommendations for ideal guidance**: Comments about ideal guidance, not specifically about the HPA guidance but generally

**Objective H - Are GPs aware of HPA and CKS guidance?**

1. **CKS guidance awareness**: Is the GP aware/not aware of CKS guidance as a whole and specifically about stool specimen microbiology, has the GP used the CKS guidance or not, include whether they are aware of/not aware of the ‘prodigy’ guidance.
2. **HPA guidance awareness**: Is the GP aware/not aware of HPA guidance as a whole and specifically about guidance on stool specimen microbiology, has the GP used the HPA guidance or not

**Objective I - What are GPs opinions on the content and layout of the HPA guidance?**

1. **Positive response to HPA guidance after review:** After the GP has reviewed the HPA guidance positive views on content, quality, layout, good points at first impression, would they use it, would they recommended it
2. **Suggested improvements to the HPA guidance:** After the GP has reviewed the HPA guidance critique on the content, quality, layout, bad points at first impression, barriers to use, suggested improvements
